# Dihydroceramide Desaturase Functions as an Inducer and Rectifier of Apoptosis: Effect of Retinol Derivatives, Antioxidants and Phenolic Compounds

**DOI:** 10.1007/s12013-021-00990-1

**Published:** 2021-05-15

**Authors:** Mariam Alsanafi, Ryan D. R. Brown, Jeongah Oh, David R. Adams, Federico Torta, Nigel J. Pyne, Susan Pyne

**Affiliations:** 1 Strathclyde Institute of Pharmacy and Biomedical Sciences, University of Strathclyde, Glasgow, Scotland UK; 2 SLING, Singapore Lipidomics Incubator, Life Sciences Institute and Department of Biochemistry, YLL School of Medicine, National University of Singapore, Singapore, Singapore; 3 School of Engineering & Physical Sciences, Heriot-Watt University, Edinburgh, UK

**Keywords:** Dihydroceramide desaturase, Polubiquitination, Fenretinide, Apoptosis

## Abstract

Dihydroceramide desaturase (Degs1) catalyses the introduction of a 4,5-*trans* double bond into dihydroceramide to form ceramide. We show here that Degs1 is polyubiquitinated in response to retinol derivatives, phenolic compounds or anti-oxidants in HEK293T cells. The functional predominance of native versus polyubiquitinated forms of Degs1 appears to govern cytotoxicity. Therefore, 4-HPR or celecoxib appear to stimulate the de novo ceramide pathway (with the exception of C24:0 ceramide), using native Degs1, and thereby promote PARP cleavage and LC3B-I/II processing (autophagy/apoptosis). The ubiquitin-proteasomal degradation of Degs1 is positively linked to cell survival via XBP-1s and results in a concomitant increase in dihydroceramides and a decrease in C24:0 ceramide levels. However, in the case of 4-HPR or celecoxib, the native form of Degs1 functionally predominates, such that the apoptotic programme is sustained. In contrast, 4-HPA or AM404 do not produce apoptotic ceramide, using native Degs1, but do promote a rectifier function to induce ubiquitin-proteasomal degradation of Degs1 and are not cytotoxic. Therefore, Degs1 appears to function both as an ‘inducer’ and ‘rectifier’ of apoptosis in response to chemical cellular stress, the dynamic balance for which is dependent on the nature of chemical stress, thereby determining cytotoxicity. The de novo synthesis of ceramide or the ubiquitin-proteasomal degradation of Degs1 in response to anti-oxidants, retinol derivatives and phenolic compounds appear to involve sensors, and for rectifier function, this might be Degs1 itself.

## Introduction

Serine palmitoyltransferase catalyses the condensation of serine and palmitoyl CoA to form 3-ketosphinganine, the rate limiting step in the de novo synthesis of ceramide [1]. 3-Ketosphinganine is converted into sphinganine, by 3-ketosphinganine reductase, which is then acylated by ceramide synthases (CerS) to form dihydroceramide. Dihydroceramide desaturase (Degs1) catalyses the introduction of a *trans* 4,5 double bond to form ceramide. The hydrolysis of sphingomyelin catalysed by sphingomyelinase is an additional route for the synthesis of ceramide. Dihydroceramide has different biological functions compared with ceramide, such as autophagy [2–4] and induction of endoplasmic reticulum (ER) stress [4], although the effect of certain Degs1 inhibitors on autophagy occur independently of dihydroceramides [5]. Significant advances in understanding the function of Degs1 have come from genetic knockout studies. Indeed, Degs1 deficiency in mouse embryonic fibroblasts is linked to increased dihydroceramide levels and AKT/protein kinase B (PKB) signalling that opposes apoptosis [6]. In addition, *Degs1*^−/−^ cells exhibit high levels of autophagy as a result of impaired ATP synthesis and activation of AMP-activated protein kinase (AMPK). Therefore, Degs1 deficiency is associated with both anabolic and catabolic pathways. We have previously proposed that these opposing effects might involve native and polyubiquitinated forms of Degs1 [7, 8]. Thus, native Degs1 is involved in induction of ceramide-dependent apoptosis that might be linked with the inhibition of pro-survival AKT signalling. In contrast, polyubiquitinated forms of Degs1 confer pro-survival signaling via activation of p38 MAPK and XBP-1s in HEK293T cells and inhibition of autophagy [8]. Other studies have shown that inhibition of Degs1 promotes cell death, which may involve autophagy or apoptosis [4, 9].

Fenretinide (*N*-(4-hydroxyphenyl)retinamide, 4-HPR) is a synthetic retinoid that is being evaluated as an anti-cancer agent for breast [10, 11], ovarian [12], neuroblastoma [13], lung [14] and other [15] cancers. 4-HPR has been characterised as a competitive irreversible inhibitor of Degs1 [16], which blocks ceramide accumulation in liver and skeletal muscle and reduces peripheral insulin resistance and hepatic steatosis in DIO mice [17]. 4-HPR also increases adiponectin and resistin levels and decreases retinol binding protein 4 (RBP4) and leptin levels, thereby suggesting that it increases adipogenesis [18]. However, this contrasts with the use of other Degs1 inhibitors, such as GT-11, which blocks rosiglitazone (PPARγ agonist)-induced differentiation of adipocytes [19] and this is supported by the finding that dihydroceramides, which accumulate upon inhibition of Degs1, reduce differentiation of adipocytes. The mechanism of action of GT-11 does not recapitulate the genetic loss of Degs1, as AKT or AMPK signaling in response to insulin is unaltered. The effect of GT-11 is also different from 4-HPR, which does not block rosiglitazone-induced differentiation of adipocytes. 4-HPR also normalises mitochondrial metabolism with low TCA cycle intermediate and oxidative stress marker levels in white adipocytes of HFD-fed mice. However, in common with the *Degs1*^−/−^, 4-HPR promotes autophagy of mature adipocytes [6]. The use of the metabolite of 4-HPR, 4-oxo-*N*-(4-hydroxyphenyl)retinamide (termed 4-OXO) supports these findings [20]. Thus, 4-OXO fails to inhibit adipogenesis [20] and in fact, increases the expression of adipogenic markers. 4-OXO also stimulates phosphorylation of AKT and autophagy that is not related to the regulation of retinol gene expression [20]. Thus, GT-11 and 4-HPR have distinct mechanisms of action on Degs1. We have suggested that the difference might be a consequence of the ability of 4-HPR, but not GT-11, to promote the polyubiquitination of Degs1 [8].

In the current study, we have investigated the role of Degs1 in chemical-induced cellular stress in HEK293T cells treated with retinol analogues, phenolic compounds and anti-oxidants [21]. This was undertaken in order to define the role of native versus polyubiquitinated Degs1 forms in mediating cytotoxic effects of some of these agents.

## Materials and Methods

### Materials

All general biochemicals were from Sigma (Gillingham, UK) including MG132 (# C2211) and 4-HPA (# S510297) whereas 4-HPR (# HY-15373) was from Fisher Scientific (Leicester, UK). AM404 (# CAY90060), acetaminophen (# CAY10024), celecoxib (# CAY10008672), γ-tocopherol (# CAY27193), γ-tocotrienol (# CAY10008494), phenoxodiol (# HY-13721) and resveratrol (# CAY70675) were all from Cambridge Bioscience (Cambridge, UK). GT-11 (# 857395) was from Avanti Polar Lipids (Alabama, USA).

Anti-GAPDH (# sc-47724) antibody was from Insight Biotechnology Ltd (Wembley, UK); anti-Degs1 (# ab185237) antibody was from Abcam; anti-PARP (# 9542) and anti-XBP-1s (# 12782) antibodies were from New England Biolabs Ltd. (Hitchin, UK). DharmaFECT^TM^ reagent, ON-TARGETplus SMARTpool^®^ and Degs1 siRNAs were from Dharmacon (Cromlington, UK). Scrambled siRNA (ALLSTARS Negative control) was from Qiagen (Crawley, UK).

### Cell Culture and siRNA Transfection

HEK293T cells were maintained in DMEM/Glutamax supplemented with 100 U/mL penicillin, 100 μg/mL streptomycin and 10% (v/v) foetal bovine serum at 37 °C with 5% CO_2_ before treatment with compounds. HEK293T cells were transiently transfected with siRNA constructs or scrambled siRNA (as a negative control) at a final concentration of 100 nM. Cells were transfected at ~50–60% confluence and maintained in the transfection mixture for 48 h.

### Western Blotting

Upon treatment, HEK293T cells were lysed in sample buffer containing 62.5 mM Tris-HCl (pH 6.7), 0.5 mM sodium pyrophosphate, 1.25 mM EDTA, 1.25% (w/v) sodium dodecyl sulphate, 0.06% (w/v) bromophenol blue, 12.5% (v/v) glycerol and 50 mM dithiothreitol. Proteins were separated on a 10% (v/v) acrylamide/bisacrylamide gel, and transferred to nitrocellulose Hybond membrane (GE Healthcare). Membranes were blocked in 5% (w/v) BSA (Fisher) in TBST buffer containing 20 mM Tris-HCl (pH 7.5), 48 mM NaCl, 0.1% (v/v) Tween 20 for 1 h at room temperature prior to incubation with primary antibody (diluted in blocking buffer) overnight at 4 °C. Following three washes in TBST, membranes were incubated with horse radish peroxidase conjugated anti-mouse or anti-rabbit IgG secondary antibody, as required, for 1 h at room temperature. Immunoreactive protein bands were visualised using enhanced chemiluminescence.

### Sample Preparation for Lipidomics

HEK293T cells were treated with vehicle (DMSO, 0.1% v/v final), and 4-HPR (10 μM), 4-HPA (10 μM), resveratrol (100 μM), γ-tocotrienol (35 μM), γ-tocopherol (50 μM), phenoxodiol (50 μM) or celecoxib (100 μM) for 24 h, then carefully rinsed twice with 1 mL ice cold PBS before being scraped into ice cold PBS. Cells were pelleted by centrifugation (180 *g*, 4 °C, 3 min) and the supernatant carefully removed. The cell pellet was snap frozen in liquid nitrogen for 5 sec before being stored in −80 °C for sphingolipid analysis.

### Lipidomic Analysis

A single-phase butanol:methanol (1:1, BuMe) lipid extraction protocol was used to extract the sphingolipids from cells. Cell pellets were mixed with 400 µL of BuMe and 200 µL of internal standard (ISTD) solution containing dihydroceramide d18:0/C8:0, ceramide d18:1/C8:0 and sphingomyelin d18:1/C12:0. Samples were mixed with a vortex for 10 sec and sonicated in an ultrasonic bath for 30 min on ice. Samples were then centrifuged at 14,000 g for 10 min at room temperature. The supernatants were transferred to the MS vial for LC-MS analysis.

A targeted sphingolipidomic analysis was performed to quantify dihydroceramides, ceramides and sphingomyelins. The chromatographic separation was performed on an Agilent ZORBAX Eclipse plus Rapid Resolution HD C18 (95 Å, 2.1 × 100 mm, 1.8 µm) column, at 40 °C at 400 µL min^−1^ in an Agilent 1290 UHPLC system, where the mobile phase A was 10 mM ammonium acetate, 0.2% formic acid in methanol-water (60:40, v/v) and the mobile phase B was 10 mM ammonium acetate, 0.2% formic acid in methanol-isopropanol (60:40, v/v). The gradient was set as 10% B from 0 to 3.0 min, 40% B from 3.0 to 5.0 min, 55% B from 5.0 to 5.3 min, 60% B from 5.3 to 8.0 min, 80% B from 8.0 to 10.5 min, 90% B from 10.5 to 19.0 min, 100% B from 19.0 to 22.0 min and 0% B from 22.0 to 25.0 min. The analysis was performed on an Agilent 6495 QQQ mass spectrometer (Agilent Technologies, Santa Clara, USA). The AJS ESI source parameters were the following: dry gas temperature and flow were 200 °C and 15 L min^−1^ respectively, nebulizer pressure 25 psi, sheath gas temperature and flow were set to 200 °C and 12 L min^−1^ respectively, capillary voltage and nozzle voltage set to 3500 V and 500 V respectively, and the delta EMV was 200 V. Positive high/low pressure RF was set to 210/110 and negative high/low pressure RF was 150/60. Retention time windows were set to 1 min and the cycle time to 750 ms. The fragmentor and cell acceleration voltage were set to 166 V and 25 V, respectively. The collision energy varied according to the sphingolipid. Three µL of extract were injected for analysis. The MS was operated in positive ionization mode and a dynamic multiple reaction monitoring method (dMRM) was used for the analysis. For each SP molecular species, the MRM transition with the highest response was used as a quantifier and the transition with a lower response was used as a qualifier. However, in certain low abundant lipids, and some molecular species of the SM class, the peaks were identified only based on retention times and the quantifier, as the signal of the qualifier was not detectable. The fragmentation patterns of SP have been previously described. For ceramides and dihydroceramides the product ion corresponds to the characteristic SPH. For d18:1 and d18:0 the product ions are *m/z* 264 and 284, respectively. SPH product ions can be generated from either intact precursor [M + H]^+^ or the precursors that underwent a water loss [M-H_2_O + H]^+^ in the ion source. For sphingomyelins (SM), *m/z* 184.1 corresponding to the phosphocholine was used as a quantifier and SPH product ion was used as a qualifier. Signals were considered for further analysis if showing a CV < 20%, a S/N > 10 and a linear variation in a dilution curve.

### Data Analysis

The acquired MS data were analyzed using Agilent MassHunter software version B.08.00. The signal to noise ratios (S/N) were calculated using the raw peak areas in the study samples and processed blanks (PBLK). Lipids that had S/N < 10 and a CoV > 30% (calculated from a pooled QC analysed every 5 samples) were discarded. Internal standards (IS) were used to normalize the raw peak areas using corresponding quantifier/qualifier transitions. The values after normalization to IS were further normalized to the protein amount in each sample.

### Densitometry

Quantification of immuno-reactive bands was performed using ImageJ and results expressed as the % of control for the protein of interest/GAPDH for at least *n* = 3 experiments and presented as means ± SEM.

## Results and Discussion

We have previously demonstrated that the sphingosine kinase 1 (SK1) inhibitor, SKi and the Degs1 inhibitor, 4-HPR induce the ubiquitin-proteasomal degradation of Degs1 in HEK293T cells [7]. Others have used phenolic compounds and anti-oxidants to demonstrate Degs1 inhibitors promote autophagy in T98G and U87MG glioblastoma cells [5]. These compounds include resveratrol, γ-tocotrienol, γ-tocopherol, phenoxodiol and celecoxib. We have investigated these compounds using HEK293T cells at concentrations that have previously been shown to increase dihydroceramide levels and to induce autophagy in glioblastoma cells [5].

### Sphingolipid Levels

**Fig. 1:**
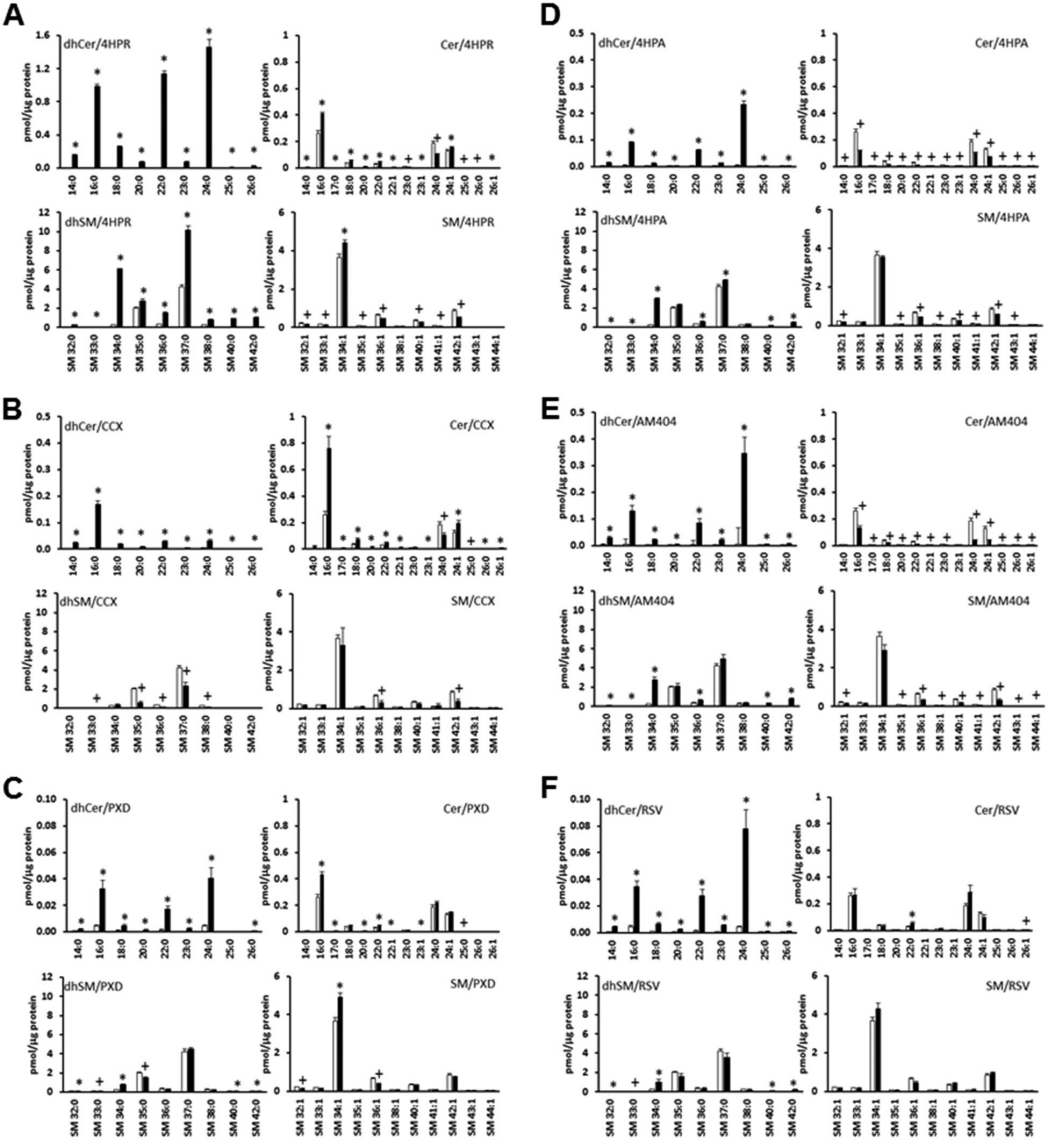
Sphingolipid levels. Cells maintained in serum and grown to 70% confluence were treated with (**A**) 4-HPR (10 μM) or (**B**) or celecoxib (CCX, 100 μM) or (**C**) phenoxodiol (PXD, 10 μM) or (**D**) 4-HPA (10 μM) or (**E**) AM404 (10 μM) or (**F**) resveratrol (RSV, 100 μM) for 24 h before snap-freezing. Lipid extracts were analysed by LC-MS for different molecular species of ceramide (upper right panel), dihydroceramide (upper left panel), sphingomyelin (lower right panel) and dihydrosphingomyelin (lower left panel) levels. The *x* axis annotates different N-acyl chain lengths and double bond molecular species. Results are *n* = 3 independent samples and are expressed as means ± SEM. *p < 0.05 increase in treated vs. control; ^+^*p* < 0.05 decrease in treated vs. control (unpaired *t* test)

The treatment of HEK293T cells with 4-HPR, celecoxib or phenoxodiol increased ceramide and dihydroceramide levels in HEK293T cells. (Fig. 1A–C). The exception is C24:0 ceramide, which is decreased in response to 4-HPR (Fig. 1A) and celecoxib (Fig. 1B). All the molecular species with different acyl chain lengths are modulated but there are differences in the relative abundance with C16:0, C22:0 and C24:0 dihydroceramides and C16:0, C24:0 and C24:1 ceramides being the major species (Fig. 1A-C). The increase in dihydroceramide levels in response to 4-HPR is consistent with elevated levels of dihydrosphingomyelin species, that are synthesised from dihydroceramides (Fig. 1A). The increase in C16:0 ceramide in response to 4-HPR is also associated with an elevation in the corresponding C34:1 (d18:1/16:0) sphingomyelin (Fig. 1A), while the decrease in C24:0 ceramide is associated with a reduction in C42:1 (d18:1/24:0) sphingomyelin levels (Fig. 1A). The levels of dihydroceramide formed in response to celecoxib or phenoxodiol were low and therefore changes in dihydrosphingomyelin or sphingomyelin are also modest at best (Fig. 1B, C). 4-HPA and AM404 increased dihydroceramide levels and reduced ceramides (including C24:0 ceramide) (Fig. 1D, E). With 4-HPA, the most abundant species are C16:0, C22:0 and C24:0 dihydroceramides and C16:0, C18:0, C24:0 and C24:1 ceramides (Fig. 1D). The changes in dihydroceramides/ceramides are consistent with increases in dihydrosphingomyelin species and a reduction in sphingomyelin species levels (Fig. 1D, E). Resveratrol increased dihydroceramide levels (albeit the levels are low) but did not affect ceramides levels (with the exception of C22:0 ceramide, which is low) (Fig. 1F). Therefore, consequential effects on dihydrosphingomyelin and sphingomyelin are minimal (Fig. 1F). The differential effect on ceramides/dihydroceramides of these compounds was further explored by assessing their impact on the polyubiquitination of Degs1, PARP cleavage (marker of apoptosis) and LC3B-I/II (marker of autophagy). This was undertaken in order to more precisely define the role of Degs1 in the cytotoxic mechanism of action of some of these compounds in HEK293T cells.

### 4-HPR Induces the Polyubiquitination of Degs1

**Fig. 2:**
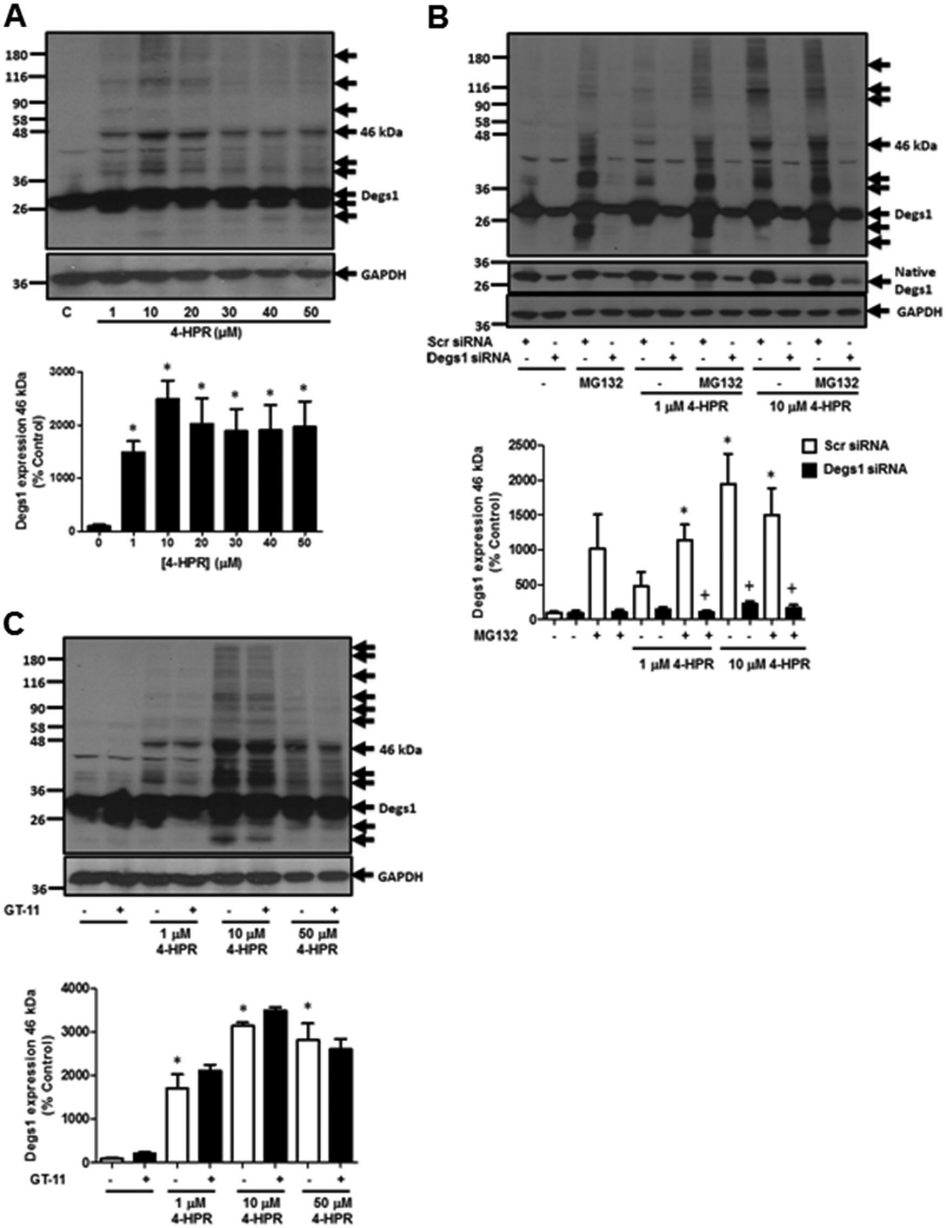
The effect of 4-HPR on Degs1 protein laddering in HEK293T cells. Cells maintained in serum and grown to 70% confluence were treated with 4-HPR (1–50 μM) for 24 h. In certain cases, cells were treated with scrambled or Degs1 siRNA (100 nM) for 48 h or MG132 (10 μM) or GT-11 (10 μM) for 30 min prior to 4-HPR. **A** Western blot probed with anti-Degs1 antibody showing the concentration-dependent effect of 4-HPR on Degs1 laddering. **B** Western blot probed with anti-Degs1 antibody showing the effect of Degs1 siRNA on formation of the Degs1 ladder in response to 4-HPR (1 and 10 μM) or MG132. **C** Western blot probed with anti-Degs1 antibody showing the lack of effect of GT-11 on 4-HPR stimulated formation of the Degs1 ladder. Blots were re-probed for GAPDH using anti-GAPDH antibody to ensure comparable protein loading. The blot is from a single experiment, representative of at least three independent experiments. Also shown are bar graphs of densitometric data (measuring the 46 kDa ubiquitinated Degs 1; expressed as means ± SEM) using combined data from at least three independent experiments. *p < 0.05 treated vs. control (one way ANOVA and Dunnett’s post-test); ^+^*p* < 0.05 Degs1 siRNA-treated vs. corresponding scr siRNA-treated (one way ANOVA and Bonferroni’s post-test)

First, we investigated the effect of 4-HPR on Degs1. Native Degs1 is expressed as a 32 kDa protein in HEK293T cells, detected with an anti-Degs1 antibody on western blots (Fig. 2A). Treatment of HEK293T cells with 4-HPR (1–50 μM, 24 h) induced the appearance of a ladder of higher molecular mass protein bands that immuno-reacted with anti-Degs1 antibody, including proteins with Mr = 36 and 46 kDa (Fig. 2A). We have previously shown that the ladder is composed of polyubiquitinated forms of Degs1 [7]. The formation of the ladder decreases at concentrations of 4-HPR that are above 20 μM (Fig. 2A), indicating that the rate of degradation exceeds the rate of de novo synthesis of Degs1. Confirmation of the identity of the higher molecular mass Degs1 forms was established using siRNA to knockdown Degs1 expression, which reduced the immuno-reactive intensity of the 32 kDa protein and the laddered protein bands (Fig. 2B). The dynamic nature of the turnover of Degs1 is indicated by the finding that the treatment of HEK293T cells with the proteasome inhibitor, MG132 induced the accumulation of a similar Degs1 ladder (Fig. 2B) that was also reduced by Degs1 siRNA (Fig. 2B). Anti-Degs1 immuno-reactive bands with molecular mass < 32 kDa proteins were also detected in cells treated with MG132 (Fig. 2B), and these are likely to be forms that have been processed by the proteasome, but are captured when the chymotrypsin-like activity of the proteasome is inhibited by MG132, such that further processing is ablated. Therefore, we propose that the ability of 4-HPR to increase dihydroceramide levels in HEK293T cells might be, in part, a consequence of the ubiquitin-proteasomal degradation of Degs1. We also tested the Degs1 inhibitor, GT-11, which failed to induce the polyubiquitination of Degs1 and did not affect the response to 4-HPR (Fig. 2C), suggesting that 4-HPR might induce ubiquitin-proteasomal degradation of Degs1 by acting on an unidentified sensor that translates the chemical stress into increased dihydroceramide levels.

### Effect of 4-HPR on PARP Cleavage, Autophagy and XBP-1s Formation

**Fig. 3:**
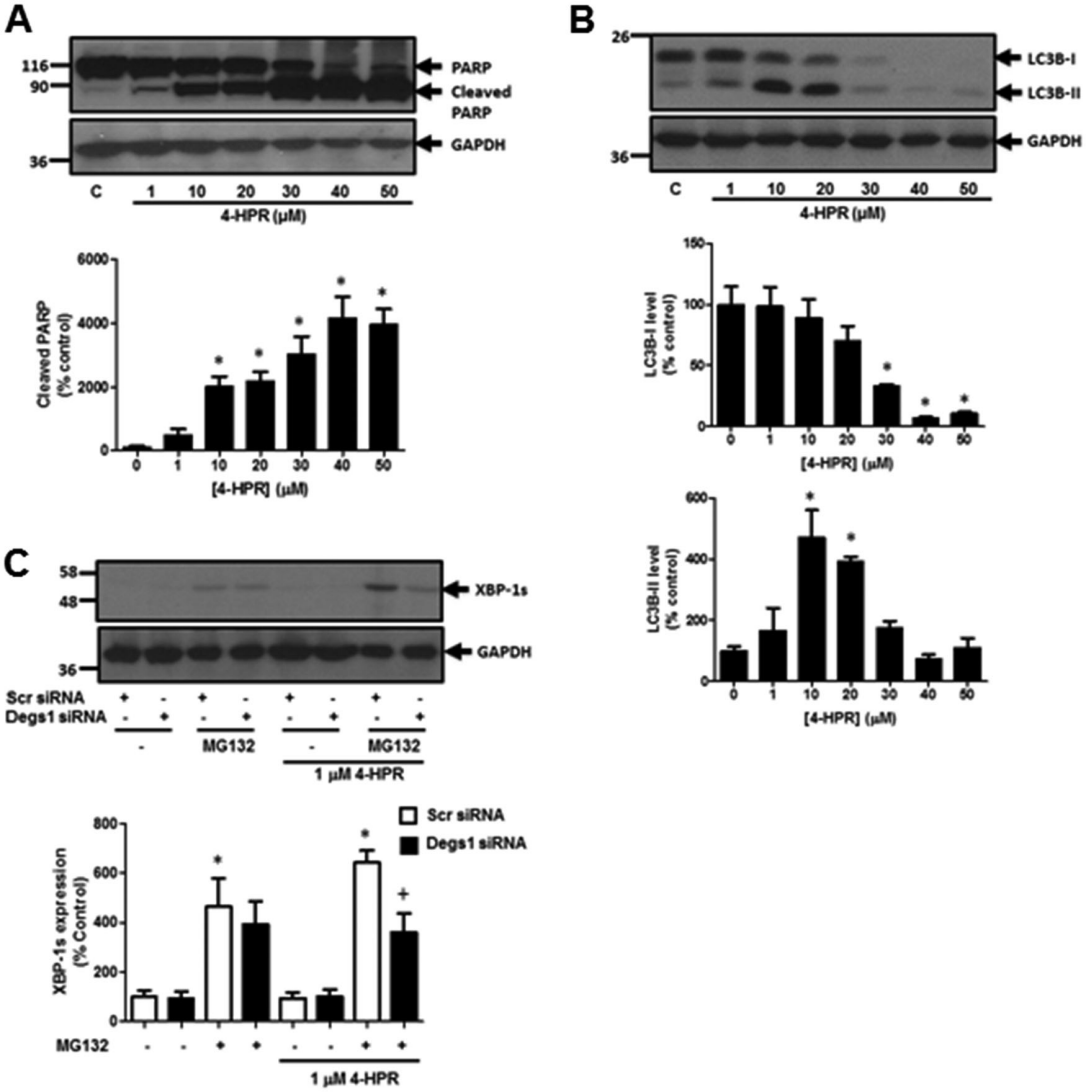
Effect of 4-HPR on PARP cleavage, LC3B-I/II and XBP-1s. Cells maintained in serum and grown to 70% confluence were treated with 4-HPR (1–50 μM) for 24 h. Cells were also treated with scrambled or Degs1 siRNA (100 nM) for 48 h or MG132 (10 μM) for 30 min. **A** Western blot probed with anti-PARP antibody showing the concentration-dependent effect of 4-HPR on PARP cleavage. **B** Western blot probed with anti-LC3B antibody showing the effect of 4-HPR on the conversion of LC3B-I to LC3B-II. **C** Western blot probed with anti-XBP-1s antibody showing the effect of Degs1 siRNA on MG132/4-HPR stimulated XBP-1s expression. Blots were re-probed for GAPDH using anti-GAPDH antibody to ensure comparable protein loading. The blot is from a single experiment, representative of at least three independent experiments. Also shown are bar graphs of densitometric data (expressed as means ± SEM) using combined data from at least three independent experiments. *p < 0.05 treated vs. control (one way ANOVA and Dunnett’s post-test); ^+^*p* < 0.05 Degs1 siRNA-treated vs corresponding scr siRNA-treated (one way ANOVA and Bonferroni’s post-test)

We have previously shown that the native forms of Degs1 is involved in the induction of apoptosis in response to the sphingosine kinase 2 inhibitor, ABC294640 [7]. In contrast, the polyubiquitinated forms of Degs1 are positively linked with the activation of pro-survival proteins, p38 MAPK and XBP-1s in HEK293T cells [7]. We show here that the treatment of HEK293T cells with 4-HPR induces the proteolytic cleavage of PARP in a concentration-dependent manner (Fig. 3A), suggesting that it is a powerful apoptotic agent. Significant PARP cleavage was evident when the concentration of 4-HPR exceeded 10 μM (Fig. 3A). 4-HPR also induced a concentration-dependent loss of LC3B-I/II, indicating that the stimulation of autophagy is closely linked with apoptosis (Fig. 3B). These findings suggest that the formation of multiple ceramide species (Fig. 1) catalysed by native Degs1 might be linked with an apoptotic programme. Native Degs1 can therefore be considered an ‘inducer’ of apoptosis and is likely responsive to an unidentified sensor that increases the de novo ceramide synthesis pathway in response to 4-HPR. This mechanism of action is supported by studies with the synthetic retinoid ST1926, which induced de novo synthesis of ceramide by activation of ceramide synthase (CerS) in malignant T cells, resulting in the death of these cells [22].

We also investigated the effect of 4-HPR on XBP-1s expression. In this regard, we used 4-HPR in combination with MG132 in order to trap the polyubiquitinated forms of Degs1. In this case, the treatment of HEK293T cells with MG132 increased the expression of XBP-1s and this was enhanced by 4-HPR (1 μM) (Fig. 3C). The reduction of Degs1 expression by Degs1 siRNA reduces the 4-HPR-dependent increase in MG312-induced XBP-1s expression (Fig. 3C), and these results are consistent with the polyubiquitinated Degs1 forms being linked with XBP-1s and cell survival [7]. Therefore, the polyubiquitinated forms of Degs1 can be considered to have a ‘rectifier’ function in terms of being anti-apoptotic. Nevertheless, at high concentrations of 4-HPR, the native Degs1 dynamically predominates and PARP cleavage/LC3B-I/II processing prevails, possibly due to the formation of apoptotic ceramide.

### 4-HPA, Acetaminophen, AM404 and Degs1

**Fig. 4:**
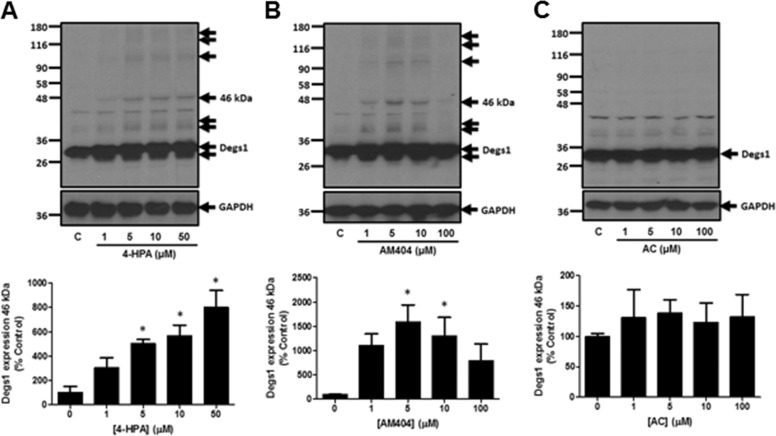
Effect of 4-HPA, AM404 and acetaminophen on Degs1 laddering. Cells maintained in serum and grown to 70% confluence were treated with (**A**) 4-HPA (1–50 μM) or (**B**) AM404 (1–100 μM) or (**C**) acetaminophen (AC, 1–100 μM) for 24 h. Western blot probed with anti-Degs1 antibody. Blots were re-probed for GAPDH using anti-GAPDH antibody to ensure comparable protein loading. The blot is from a single experiment, representative of at least three independent experiments. Also shown are bar graphs of densitometric data (measuring the 46 kDa ubiquitinated Degs 1; expressed as means ± SEM) using combined data from at least three independent experiments. *p < 0.05 treated vs. control (one way ANOVA and Dunnett’s post-test)

**Fig. 5:**
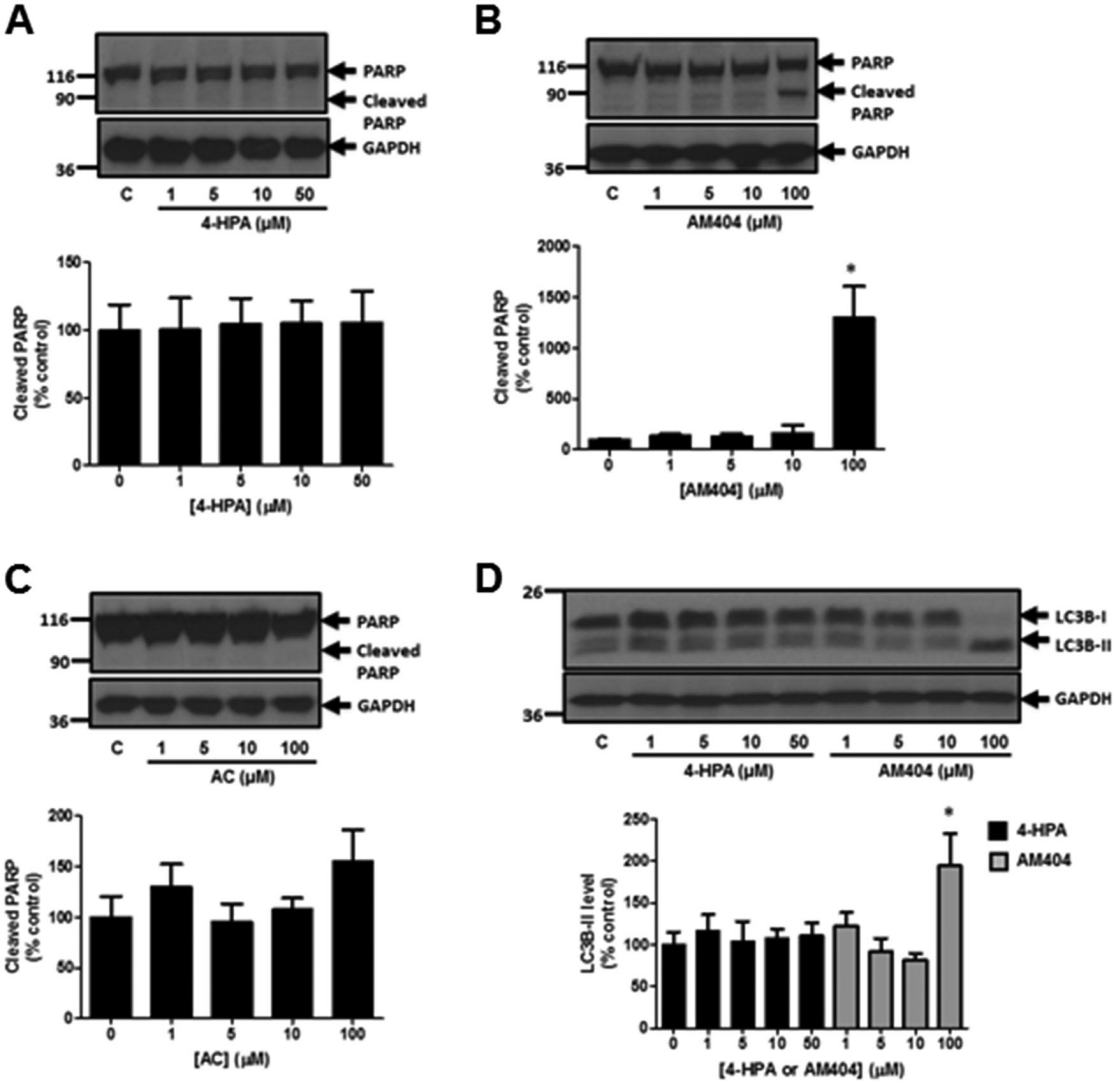
Effect of 4-HPA, AM404 and acetaminophen on PARP cleavage and LC3B-I/II processing. Cells maintained in serum and grown to 70% confluence were treated with (**A**) 4-HPA (1–50 μM) or (**B**) AM404 (1–100 μM) or (**C**) acetaminophen (AC, 1–100 μM) for 24 h. Western blot probed with anti-PARP antibody. **D** Effect of 4-HPA (1–50 μM) or AM404 (1–100 μM) on LC3B-I/II processing. Blots were re-probed for GAPDH using anti-GAPDH antibody to ensure comparable protein loading. The blot is from a single experiment, representative of at least three independent experiments. Also shown are bar graphs of densitometric data (expressed as means ± SEM) using combined data from at least three independent experiments. *p < 0.05 treated vs. control (one way ANOVA and Dunnett’s post-test)

We next assessed the effect of replacing the all-*trans*-retinoyl acyl chain in 4-HPR with other moieties, such as fatty acids in *N*-(4-hydroxyphenyl)acetamide (acetaminophen), *N*-(4-hydroxyphenyl) palmitamide(4-HPA) and *N*-(4-hydroxyphenyl)arachidonamide (AM404) on the ubiquitin-proteasomal degradation of Degs1. Since some of the compounds are derived from the metabolism of acetaminophen, the information obtained might also provide a better understanding of the pharmacological actions of these compounds, including cytotoxicity. Treatment of HEK293T cells with 4-HPA (1–50 μM) or AM404 (1–10 μM) induced the polyubiquitination of Degs1, while acetaminophen (1–100 μM) was ineffective (Fig. 4A–C). This effect of 4-HPA and AM404, which involves loss of Degs1. was associated with an increase in dihydroceramides and a reduction in ceramide levels (Fig. 1). 4-HPA, AM404 (1–10 μM) and acetaminophen failed to induce PARP cleavage (Fig. 5A–C) or LC3B-I/II processing (Fig. 5D), suggesting that compared with 4-HPR, the lack of effect of these compounds on apoptosis and autophagy might be due to their inability to use native Degs1 to form apoptotic ceramides. Instead, these agents promote only the ubiquitin-proteasomal degradation of Degs1, as evidenced by the increase in dihydroceramide and a reduction in ceramide levels. In contrast, AM404 at 100 μM induced PARP cleavage (Fig. 5B) and LC3B-I/II processing (Fig. 5D) and this might be related to actions that are Degs1-independent.

### Anti-oxidants and Degs1

**Fig. 6:**
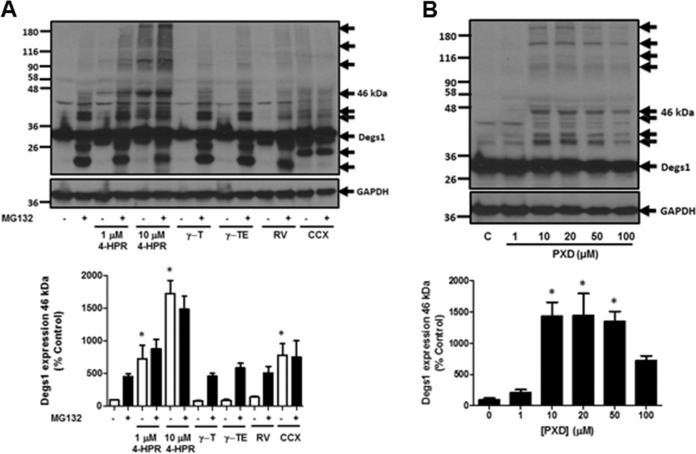
Effect of 4-HPR, resveratrol, γ-tocotrienol, γ-tocopherol, phenoxodiol and celecoxib on Degs1 laddering. Cells maintained in serum and grown to 70% confluence were treated with (**A**) 4-HPR (1 and 10 μM) or γ-tocopherol (γ-T, 50 μM) or γ-tocotrienol (γ-TE, 35 μM) or resveratrol (100 μM) or celecoxib (100 μM) or (**B**) phenoxodiol (1–100 μM) on Degs1 laddering. In some case, cells were pre-treated with MG132 (10 μM) for 30 min prior to compound. Blots were re-probed for GAPDH using anti-GAPDH antibody to ensure comparable protein loading. The blot is from a single experiment, representative of at least three independent experiments. Also shown are bar graphs of densitometric data (measuring the 46 kDa ubiquitinated Degs 1; expressed as means ± SEM) using combined data from at least three independent experiments. *p < 0.05 treated vs. control (one way ANOVA and Dunnett’s post-test)

**Fig. 7:**
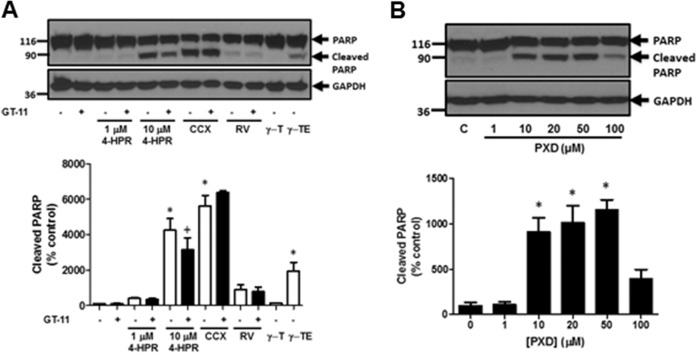
The effect of 4-HPR, resveratrol, γ−tocotrienol, γ-tocopherol, phenoxodiol and celecoxib on PARP cleavage. Cells maintained in serum and grown to 70% confluence were treated with (**A**) 4-HPR (1 and 10 μM) or γ-tocopherol (γ-T, 50 μM) or γ-tocotrienol (γ-TE, 35 μM) or resveratrol (100 μM) or celecoxib (100 μM) or (**B**) phenoxodiol (1–100 μM) on PARP cleavage. In some case, cells were pre-treated with GT-11 (10 μM) for 30 min prior to compound. Blots were re-probed for GAPDH using anti-GAPDH antibody to ensure comparable protein loading. The blot is from a single experiment, representative of at least three independent experiments. Also shown are bar graphs of densitometric data (expressed as means ± SEM) using combined data from at least three independent experiments. *p < 0.05 treated vs. control (one way ANOVA and Dunnett’s post-test); ^+^*p* < 0.05 GT-11-treated vs. corresponding treatment in absence of GT-11 (one way ANOVA and Bonferroni’s post-test)

**Fig. 8:**
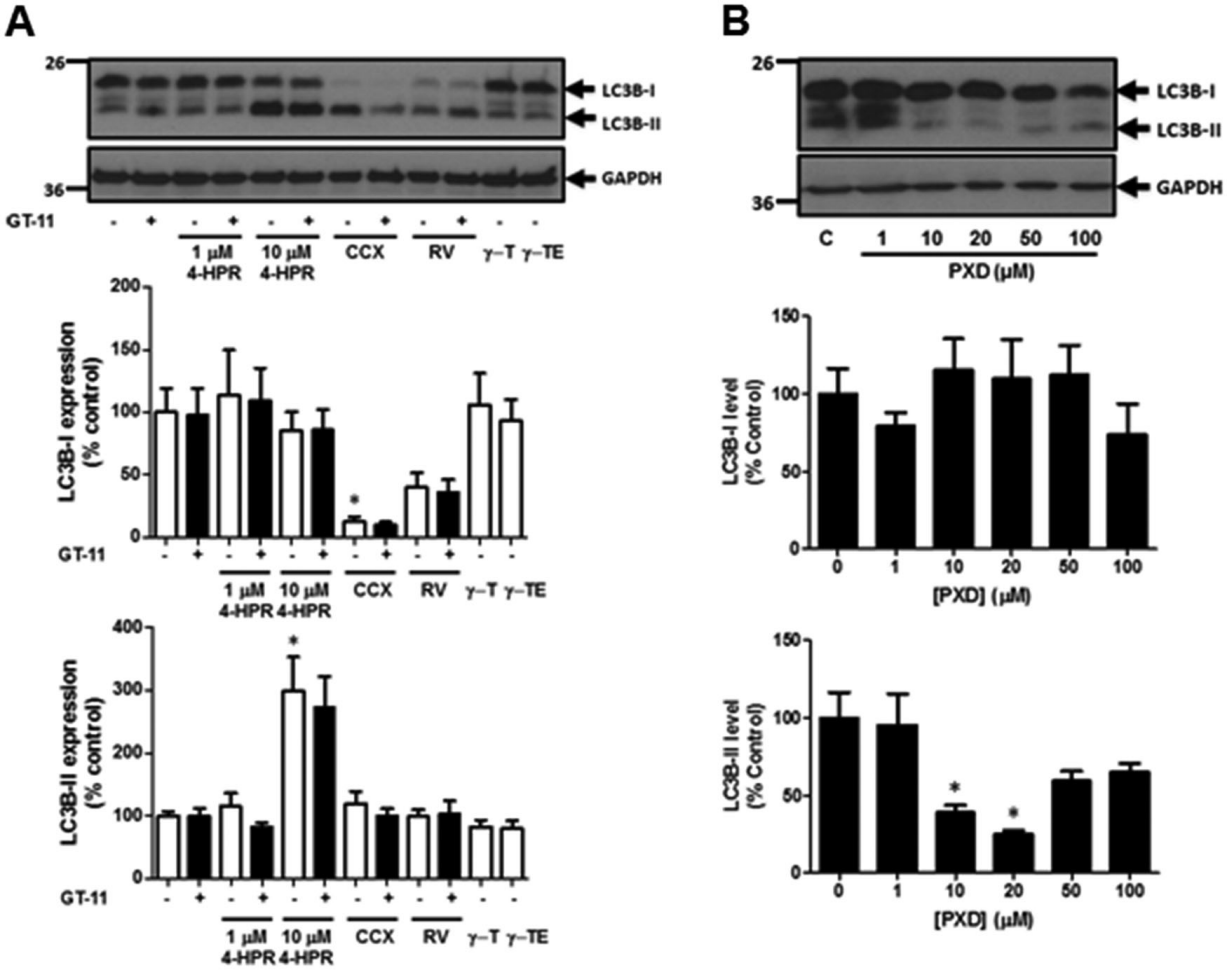
The effect of 4-HPR, resveratrol, γ−tocotrienol, γ-tocopherol, phenoxodiol and celecoxib on LC3B-I/II processing. Cells maintained in serum and grown to 70% confluence were treated with (**A**) 4-HPR (1 and 10 μM) or γ-tocopherol (γ-T, 50 μM) or γ-tocotrienol (γ-TE, 35 μM) or resveratrol (100 μM) or celecoxib (100 μM) or (**B**) phenoxodiol (1–100 μM) on LC3B-I/II processing. In some case, cells were pre-treated with GT-11 (10 μM) for 30 min prior to compound. Blots were re-probed for GAPDH using anti-GAPDH antibody to ensure comparable protein loading. The blot is from a single experiment, representative of at least three independent experiments. Also shown are bar graphs of densitometric data (expressed as means ± SEM) using combined data from at least three independent experiments. *p < 0.05 treated vs. control (one way ANOVA and Dunnett’s post-test)

Degs1 had been reported to be inhibited by a number of anti-oxidants including resveratrol (100 μM), γ-tocotrienol (35 μM), γ-tocopherol (50 μM), phenoxodiol (1–100 μM) and celecoxib (100 μM) and this was linked with autophagy in T98G and U87MG glioblastoma cells [5]. The treatment of HEK293T cells with celecoxib and phenoxodiol induced the polyubiquitination of Degs1, while resveratrol, γ-tocotrienol and γ-tocopherol were without effect (Fig. 6A, B). Celecoxib and phenoxodiol also induced PARP cleavage (Fig. 7A, B) and promoted autophagy (Fig. 8A, B), while resveratrol, γ-tocotrienol, γ-tocopherol were without a marked effect on PARP cleavage (Fig. 7A) and LC3B-I/II processing (Fig. 8A). The pre-treatment of cells with the Degs1 inhibitor, GT-11 reduced 4-HPR-stimulated PARP cleavage, suggesting a role for native Degs1 in promoting apoptosis, while it was not effective against celecoxib, probably because celecoxib provides a stronger apoptotic stimulus (Fig. 8A). Phenoxodiol and celecoxib increase ceramide (with the exception of C24:0 ceramide, which is reduced in response to celecoxib) and dihydroceramide levels (Fig. 1A, B), promote polyubiquitination of Degs1 and induce PARP cleavage and reduce LC3B-I/II levels. Therefore, celecoxib behaves similarly to 4-HPR with regard to modulating the levels of C24:0 ceramide. Acetaminophen, resveratrol and γ-tocopherol were without effect on the polyubiquitination of Degs1 (Fig. 6A) or PARP cleavage/LC3B-I/II processing (Figs. 7A, and 8A). It is notable that 4-HPR and celecoxib increase ceramide species, with the exception of C24:0 ceramide, the levels of which are decreased. These findings suggest that the ability of native Degs1 to catalyse conversion of C24:0 dihydroceramide into C24:0 ceramide might be less efficient compared with other dihydroceramide species, such that removal of Degs1 via the ubiquitin-proteasomal degradation route results in a net decrease in C24:0 ceramide levels. This contrasts with other ceramide species because the rate of their synthesis catalysed by native Degs1 appears to outpace the impact of the concomitant proteasomal degradation of Degs1 and, therefore, its use of the corresponding dihydroceramide species as substrates. Notably, γ-tocotrienol did produce a very minor effect on PARP cleavage (Fig. 7A), but failed to promote the polyubiquitination of Degs1 (Fig. 6A) and had no effect on LC3B-I/II processing (Fig. 8A). Thus, acetaminophen, resveratrol, γ-tocopherol and γ-tocotrienol do not appear to significantly modulate the ubiquitin-proteasomal degradation of Degs1 in HEK293T cells.

### Compound Structure-function Analysis

**Fig. 9:**
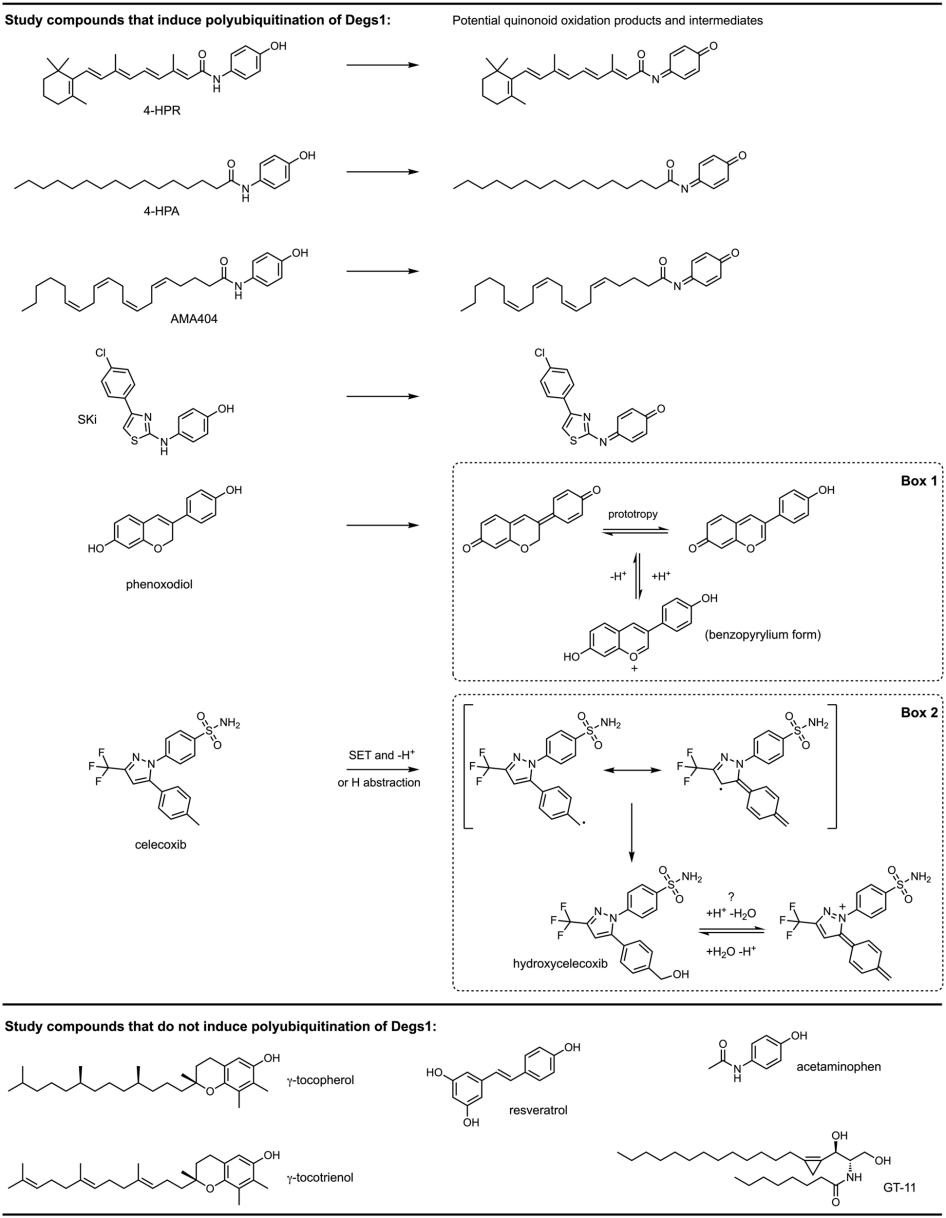
Structures of study compounds used and of putative quinonoid oxidation intermediates hypothesised to be involved in the induction of Degs1 polyubiquitination

A notable feature of commonality between the compounds that induce polyubiquitination of Degs1, and that distinguishes them from the study cohort that do not induce polyubiquitination, is the potentiality for formation of a quinonoid motif within an extended, substantially lipophilic structure. Thus 4-HPR, 4-HPA and AM404 all have potential to generate a quinone imine derivative by oxidation (Fig. 9), as does SKi, which also induces polyubiquitination of Degs1 [7]. Indeed, others, have also proposed the involvement of Degs1 in the oxidation of fenretinide/SKi to that putative quinone imine [23]. In principle, phenoxodiol also has potential to generate a quinonoid species through oxidation (Fig. 9, Box 1). In the case of celecoxib, oxidation proceeding by way of hydrogen abstraction or single electron transfer (SET) followed by deprotonation might generate a reactive intermediate radical with quinonoid character en route to hydroxycelecoxib (Fig. 9, Box 2), which is a known metabolite [24–29]. In principle, protonation and dehydration of the latter might also generate an intermediate pyrazolium species with quinone methide character (Fig. 9, Box 2). Quinone imines and related quinonoid species are redox-active entities and also have the potential to covalently modify biomolecular targets [24–29]. In this regard it is interesting that acetaminophen, which is known to generate *N*-acetyl-*p*-benzoquinone imine as a potentially toxic metabolite in vivo [30, 31], does not induce polyubiquitination of Degs1 whereas the longer chain counterpart, 4-HPA, does induce polyubiquitination. If indeed a reactive quinone imine species derived from 4-HPA is involved in the induction of Degs1 polyubiquitination, then this might suggest that a specific biomolecular ‘sensor’ target is needed for the induction mechanism, one that requires an extended lipophilic quinonoid ligand but that is essentially unresponsive to the short chain *N*-acetyl-*p*-benzoquinone imine derived from acetaminophen. According to this hypothesis there would be two key aspects to the induction of Degs1 polyubiquitination by a compound: first, a requirement for an oxidation mechanism to generate an appropriate oxidised lipophilic quinonoid entity and, second, a requirement for engagement of a ‘sensor’ target (potentially through oxidation or covalent modification). In principle, a single protein might both serve to oxidise the compound and act as the ‘sensor’ (e.g. by covalently trapping the quinonoid species in situ). Indeed, in the case of celecoxib it is difficult to conceive that a reactive quinonoid radical might be released from one protein to target another, although formation and release of hydroxycelecoxib by one protein might potentially target a second and distinct ‘sensor’ biomolecule, if the metabolite is sufficiently reactive towards dehydration so as to generate the putative quinone methide (Box 2 of Fig. 9) at the ‘sensor’. It should be noted that Degs1 itself might potentially serve to oxidise and activate the compounds that induce its own polybiquitination and degradation. The structure of Degs1 has yet to be determined, but it exhibits sequence homology to another integral membrane desaturase, namely to stearoyl-CoA desaturase 1 (SCD1), for which X-ray crystal structures have recently been obtained [32–34]. These structures reveal the presence a di-iron catalytic centre that is responsible for mediating the oxidation of substrate, which binds with its acyl chain in a bent hydrophobic canal. Thus, the oxidation of fenretinide and related compounds by the cognate di-iron centre of Degs1 should be considered as a possibility. Moreover, the potential of Degs1 itself to act as the putative ‘sensor’ (e.g. by reaction with a quinonoid intermediate generated at the enzyme) should also be considered. Indeed, it is conceivable that there might be an evolutionary advantage to the targeted degradation of Degs1 in response to the irreversible binding of a xenobiotic quinone because subsequent obstruction of dihydroceramide substrate entry to the enzyme might result in decoupling of oxygen activation from substrate oxidation. Such a situation might lead to unbridled generation of reactive oxygen species (ROS).

## Conclusion

**Fig. 10:**
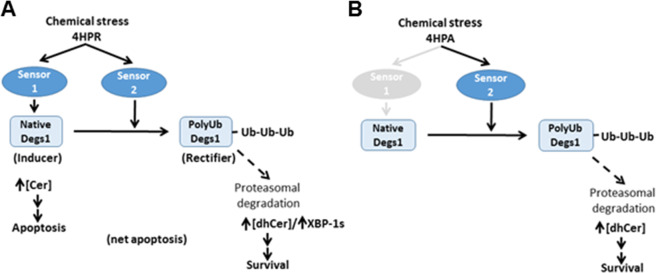
Summary of the role of Degs1 as an inducer and rectifier of apoptosis. **A** 4-HPR binds to sensor 1 to induce apoptotic ceramide formation by native Degs1. 4-HPR also binds to sensor 2 (potentially Degs1 itself) to promote rectifier function (i.e. polyubiquitination of Degs1), which has a pro-survival function and results in accumulation of dihydroceramide. The net balance of inducer and rectifier function is tilted toward the former thereby resulting in apoptosis for 4-HPR. Therefore, 4-HPR is cytotoxic. **B** 4-HPA binds to sensor 2 (potentially Degs1 itself) to induce the rectifier response (i.e. ubiquitin-proteasomal degradation of Degs1). Therefore, 4-HPA is not cytotoxic

The findings in this study are consistent with a model in which Degs1 appears to function both as an inducer (native Degs1 and apoptotic ceramide) and rectifier (ubiquitin-proteasomal degradation of Degs1 and dihydroceramide accumulation) of apoptosis in response to chemical cellular stress. The balance of the inducer/rectifier function of Degs1 is dependent on the nature of chemical stress, thereby determining cytotoxicity. Agents such as 4-HPR induce apoptosis because the dynamic balance is towards native Degs1 and therefore production of ceramide, which is higher in cells treated with 4-HPR. It remains to be determined whether there is a putative sensor that binds 4-HPR that promotes the de novo ceramide pathway. In contrast, agents tested here that do not induce apoptosis, such as 4-HPA, reduce ceramide and increase dihydroceramide levels, a consequence of the ubiquitin-proteasomal degradation of Degs1. The rectifying effect might be a consequence of polyubiquitinated Degs1 being endowed with pro-survival function via p38 MAPK activation and XBP-1s expression [7] (see schematic in Fig. 10). Further studies are required to determine whether there is a sensor that translates chemical stress into the ubiquitin-proteasomal degradation of Degs1 in response to compounds that can form a quinonoid motif within an extended, substantially lipophilic structure. In this regard, Degs1 might be the sensor itself.

Finally, our findings suggest that 4-HPA might be considered solely a Degs1 inhibitor, because it fails to promote ceramide formation and this compound could, therefore, be exploited in diseases, such as T2D, where the major therapeutic objective is to abrogate ceramide-induced metabolic deregulation and to avoid cytotoxicity. Indeed, Degs1 deficiency improves insulin resistance and hepatic steatosis [35]. In contrast, 4-HPR is likely to be better targeted to tumours, as has been the case, where cancer cell cytotoxicity is the therapeutic objective.

## Abbreviations

4-HPR: *N*-(4-hydroxyphenyl)retinamide or fenretinide
4-HPA: *N*-(4-hydroxyphenyl)palmitamide
Acetaminophen: *N*-(4-hydroxyphenyl)acetamide
AM404: *N*-(4-hydroxyphenyl)arachidonamide or *N*-(4-hydroxyphenyl)-5*Z*,8*Z*,11*Z*,14*Z*-eicosatetraenamide
CerS: ceramide synthase
Degs1: dihydroceramide desaturase
ER: endoplasmic reticulum
GT-11: *N*-[(1*R*,2*S*)-1,3-dihydroxy-1-(2-tridecylcyclopropen-1-yl)propan-2-yl]octanamide
MAPK: mitogen activated protein kinase
MG132: carbobenzoxy-Leu-Leu-leucinal
PARP: Poly(ADP-Ribose) Polymerase
SKi: 2-(*p*-hydroxyanilino)-4-(*p*-chlorophenyl)thiazole or 4-[[4-(4-chlorophenyl)-2-thiazolyl]amino]phenol
XBP-1s: X-box protein-1s
